# Behavioral Intelligence in Action: Implementation Science and Cardiovascular Learning Health Systems

**DOI:** 10.1016/j.jacadv.2025.102351

**Published:** 2025-12-03

**Authors:** Atsushi Mizuno, Faraz S. Ahmad, Darshan H. Brahmbhatt

**Affiliations:** a Department of Cardiovascular Medicine, St. Luke’s International Hospital, Tokyo, Japan; b Division of Cardiology, Department of Medicine, Northwestern University Feinberg School of Medicine, Chicago, Illinois, USA; c Center for AI, Northwestern Medicine Bluhm Cardiovascular Institute, Chicago, Illinois, USA; d Division of Cardiology, Mount Sinai Hospital, Sinai Health, Toronto, Ontario, Canada; e Ted Rogers Centre for Heart Research, Peter Munk Cardiac Centre, University Health Network, Toronto, Ontario, Canada; f Division of Cardiology, Department of Medicine, University of Toronto, Toronto, Ontario, Canada

**Figure:**
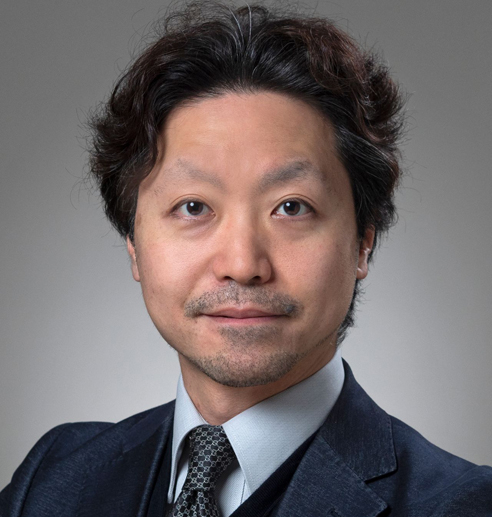

**Figure:**
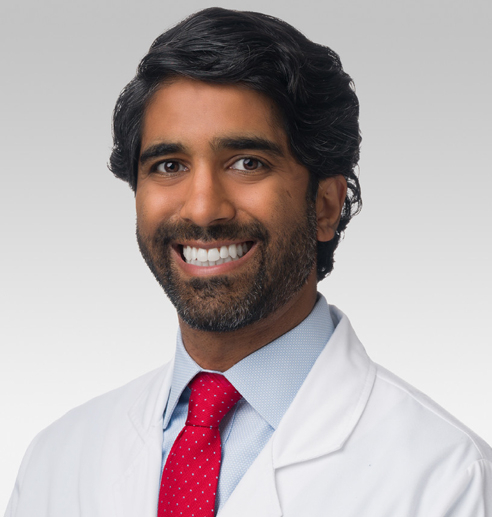

**Figure:**
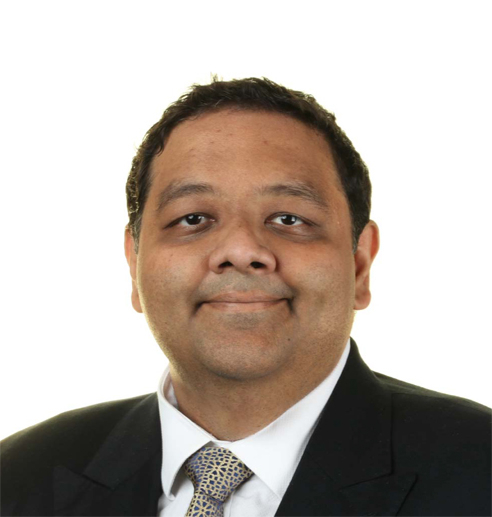

We are proud to introduce this mini-focus issue of *JACC: Advances* on Implementation Science and Cardiovascular Learning Health Systems (LHS). As cardiovascular medicine continues to evolve, the need to bridge the gap between discovery and delivery has never been more urgent. Implementation science and the LHS framework together provide powerful means to accelerate the adoption of evidence-based practices, foster continuous learning within health systems, and ultimately transform how cardiovascular care is delivered. This special issue assembles 17 contributions—including forward-looking reviews, thought-provoking viewpoints, and rigorous mixed-methods studies—that collectively aim to drive meaningful and sustainable improvements in cardiovascular outcomes worldwide. Together, these works embody a shared commitment to building a future in which evidence and practice continually inform each other, promoting better, fairer, and more resilient cardiovascular health systems.

Biswas et al^1^ deliver a comprehensive and review of digital transformation and artificial intelligence within Learning Health Systems. They conceptualize the LHS as a dynamic, self-improving infrastructure that integrates clinical care, data science, and continuous feedback loops to promote equitable cardiovascular outcomes. Their work provides a roadmap for how artificial intelligence (AI)-enabled learning cycles can accelerate discovery and enhance delivery at scale. Building on this systems-level vision, De Oliveira-Gomes et al^2^ focus on behavioral implementation strategies, particularly how nudges can be embedded within cardiovascular–kidney–metabolic care to support clinician decision-making and strengthen patient engagement. By synthesizing frameworks such as MINDSPACE and EAST, they demonstrate how subtle environmental and cognitive cues can increase the adoption of evidence-based practices—expanding the behavioral dimension of implementation science in cardiovascular medicine. Complementing these perspectives, Teow et al^3^ present a foundational conceptual review using frailty in cardiovascular care as an example. As the population being treated with cardiovascular disease becomes older, a number of competing risks from frailty gain importance in their management. By clarifying key constructs such as implementation strategies, hybrid effectiveness–implementation designs, and contextual adaptation, they create an accessible bridge for clinicians entering this evolving field. Together, these three reviews establish the conceptual backbone of this collection, offering readers a foundation from which to interpret and connect the diverse empirical and viewpoint contributions that follow.

Chen et al^4^ apply a convergent mixed-methods design to identify barriers and facilitators to implementing polypill therapy for heart failure in the United States. As noted in the accompanying editorial by Bui et al^5^ this represents an upstream phase of implementation—proactively mapping contextual challenges before large-scale deployment. In a distinct application, Johnson et al^6^ evaluate remote patient monitoring for hypertension management, demonstrating how patient- and system-level factors influence implementation success in real-world settings. Together, these investigations illustrate how mixed-methods frameworks can be leveraged both proactively—to prepare interventions for success—and analytically—to assess and refine their impact, underscoring the versatility of implementation science across the translational spectrum.

Several data-driven investigations in this issue further highlight how AI and natural language processing applied to electronic health records can advance real-world cardiovascular implementation while promoting equity and representativeness. Hassan et al^7^^,^^8^ developed a 2-stage hybrid natural language processing model to identify pulmonary embolism at scale, while Breeze et al^9^ used AI-assisted population analytics to contrast real-world cohorts with randomized controlled trial participants, revealing disparities among women, minorities, and socioeconomically disadvantaged groups. Extending these advances into diagnostics, Zhou et al^10^ validated an AI-enabled stethoscope for valvular heart disease detection, and Duffy et al^11^ externally validated a now U.S. Food and Drug Administration–cleared deep learning algorithm that can be applied to echocardiographic videos to accurately identify cardiac amyloidosis—collectively illustrating the progression of data-driven tools from population-level insight to direct clinical translation.

Other contributions in this issue demonstrate the power of implementation science in clinical domains where needs are well defined and data are structured. Venkatraman et al^12^ highlight advanced heart failure and transplantation as paradigms for bridging robust clinical data with structured care pathways. Naderi et al^13^ extend this discussion to women’s cardiovascular health, illustrating how Learning Health System approaches can help close persistent sex-based gaps in diagnosis, treatment, and follow-up. Sterling et al^14^ emphasize the infrastructure and governance required for equitable cardiovascular implementation, including home health models and informed consent frameworks for responsible digital integration. Viewpoints by Reddy et al^15^ and Sritharan et al^16^ further stress that sustainable translation of AI and digital tools requires not only innovation but also ethical stewardship and participatory design. Finally, as illustrated by Aslanger et al^17^ the emergence of implementation-aware trial protocols—such as those integrating new occlusive MI and nonocclusive MI classification or hybrid effectiveness designs—signals a forward-looking trajectory in which pragmatic science and learning systems converge to close the gap between discovery and delivery.

Ultimately, this mini-focus issue reveals how two complementary forces are converging to redefine cardiovascular implementation: the tangible power of digital and AI technologies, and the conceptual strength of behavioral and implementation frameworks. Data-driven tools and machine intelligence bring precision, scalability, and immediacy, while frameworks such as nudge theory, hybrid effectiveness–implementation designs, and LHS principles provide the structure and intentionality to transform innovation into sustainable practice. When united, these forces create a synergistic engine for transformation—one that not only accelerates the uptake of evidence but also embeds continuous learning, equity, and human-centered design at the heart of cardiovascular care. The future of cardiovascular medicine will depend on how effectively we align these tools and theories—turning knowledge into action, and innovation into impact—for the benefit of patients and health systems worldwide.

## Funding support and author disclosures

Dr Ahmad has received research support from Pfizer, Atman Health, Tempus, AstraZeneca, IDoven, Ultromics, Abiomed, and Anumana; and has received consulting fees and honoraria from AstraZeneca and Alnylam Pharmaceuticals. All other authors have reported that they have no relationships relevant to the contents of this paper to disclose.
